# Menaquinone-mediated regulation of membrane fluidity is relevant for fitness of *Listeria monocytogenes*

**DOI:** 10.1007/s00203-021-02322-6

**Published:** 2021-04-19

**Authors:** Alexander Flegler, Vanessa Kombeitz, André Lipski

**Affiliations:** Institute of Nutritional and Food Science, Food Microbiology and Hygiene, University of Bonn, Friedrich-Hirzebruch-Allee 7, 53115 Bonn, Germany

**Keywords:** *Listeria monocytogenes*, Cold adaptation, Fatty acids, Menaquinone, Membrane fluidity, Bacterial cell fitness

## Abstract

*Listeria monocytogenes* is a food-borne pathogen with the ability to grow at low temperatures down to − 0.4 °C. Maintaining cytoplasmic membrane fluidity by changing the lipid membrane composition is important during growth at low temperatures. In *Listeria monocytogenes*, the dominant adaptation effect is the fluidization of the membrane by shortening of fatty acid chain length. In some strains, however, an additional response is the increase in menaquinone content during growth at low temperatures. The increase of this neutral lipid leads to fluidization of the membrane and thus represents a mechanism that is complementary to the fatty acid-mediated modification of membrane fluidity. This study demonstrated that the reduction of menaquinone content for *Listeria monocytogenes* strains resulted in significantly lower resistance to temperature stress and lower growth rates compared to unaffected control cultures after growth at 6 °C. Menaquinone content was reduced by supplementation with aromatic amino acids, which led to a feedback inhibition of the menaquinone synthesis. Menaquinone-reduced *Listeria monocytogenes* strains showed reduced bacterial cell fitness. This confirmed the adaptive function of menaquinones for growth at low temperatures of this pathogen.

## Introduction

*Listeria monocytogenes* (*L*. *monocytogenes*) is a Gram-positive, facultative anaerobic bacterium that is responsible for the foodborne disease listeriosis. In 2018, the European Food Safety Authority (EFSA) identified listeriosis as one of the most severe zoonoses with the highest case fatality rate of 15.6% based on severity data (EFSA-ECDC 2019). The ability of *L*. *monocytogenes* to grow at low temperatures allows this bacterium to persist in food processing plants, which can lead to the contamination of food and thus to the spread of listeriosis (Lopez-Valladares et al. 2018). *L*. *monocytogenes* can grow in a temperature range from − 0.4 to 50 °C (Farber and Peterkin 1991; Walker et al. 1990). For that reason, the capacity of *L*. *monocytogenes* to grow at low temperatures is crucial for its ability to colonize, reproduce and persist in the food-processing environment and on food-processing equipment (Ryser and Marth 2007).

One of the most important adaptations to low growth temperatures is the regulation of membrane fluidity (Gounot and Russell 1999; Mykytczuk et al. 2007; Suutari and Laakso 1994; Zhang and Rock 2008). The function of the cell membrane depends on the physical state of the lipid bilayer. To provide an ideal setting for membrane-associated cell functions, a lipid bilayer must be in a liquid-crystalline state (Mendoza and Cronan 1983). A decrease in membrane fluidity and the associated phase transition to a solid, gel-like state leads to an impairment of growth (Annous et al. 1997; Chihib et al. 2003; Jones et al. 2002). Therefore, the regulation of the membrane fluidity ensures the biologically active state of the membrane and enables the bacterial cells to adapt to varying environmental temperatures. Generally, a decrease in the temperature slows down the reaction rates of various cellular processes and also reduces the fluidity of the bacterial cell membrane (Tasara and Stephan 2006). The decrease of the membrane fluidity could disrupt membrane-associated processes such as electron transport in the respiratory chain, membrane permeability and substrate transport (Zhang and Rock 2008). Moreover, lower reaction rates also result in lower growth rates. To avoid those disruptions, bacteria modulate membrane fluidity by modifying their fatty acid composition. To prevent liquid-gel transition at low temperatures, fatty acids with lower melting temperatures are incorporated into the membrane. The fluidity of biological membranes is mainly determined by the lipid-acyl chains of polar lipids (Russel 1984). The cytoplasmic membrane of *L*. *monocytogenes* consists mainly of the branched fatty acids *anteiso*-C_15:0_ and *anteiso*-C_17:0_. When growing under cold conditions, the proportion of *anteiso*-C_17:0_ decreases and the proportion of *anteiso*-C_15:0_ increases until the latter accounts for up to 80% of the total fatty acid profile (Annous et al. 1997; Mastronicolis et al. 1998, 2006; Tatituri et al. 2015). This causes the membrane fluidity to be maintained since the fatty acid *anteiso*-C_15:0_ has a lower melting point (Knothe and Dunn 2009). Several strains of *L*. *monocytogenes* show an additional mechanism for the regulation of membrane fluidity. In a previous study, it was shown that besides fatty acid modification, the menaquinone-7 (MK-7) content also contributes to the regulation of membrane fluidity in *Listeria* by broadening the phase transition (Seel et al. 2018). Similar effects were previously shown for various artificial systems in lipid vesicles mixed with ubiquinone-3, vitamin K_1_, and cholesterol (Asai 2000; Asai and Watanabe 1999; Harris et al. 2002; Ortiz and Aranda 1999; Seel et al. 2018). Quinones are ubiquitous lipid-soluble molecules that are mainly known for their involvement in membrane-bound electron transport in the respiratory chain and part of the cytoplasmic membrane (Søballe and Poole 1999). Low temperatures of 6 °C induce an increased production of MK-7 in some *L*. *monocytogenes* strains, thus expanding the phase transition to a gel-like membrane state to maintain the membrane fluidity under low- temperature growth conditions. The synthesis of MK-7 could be a second important cold adaptation mechanism due to the less energy requirement compared to the required energy for the fatty acid synthesis (Seel et al. 2018; Zhang and Rock 2008). However, it is still unknown how the suppression of the thermotropic phase transition by higher amounts of menaquinones (MKs) has an impact on membrane integrity and finally on bacterial cell fitness under low-temperature conditions. Using the term fitness as a quantitative attribute for the survival of an external stressor, this study tested the fitness of bacterial cells by subjecting them to freeze–thaw stress. Resistance to freeze–thaw stress is an accepted indicator for cell membrane integrity and bacterial cell fitness (Carlquist et al. 2012; Sleight et al. 2006). It was hypothesised that supplementation with inhibitors for MK synthesis will cause negative effects on membrane integrity and finally on resistance to temperature stress and growth rates of *L. monocytogenes* strains under low-temperature conditions. In this project, aromatic amino acids (AAA) were used as inhibitors for MK synthesis. Tsukamoto et al. (2001) demonstrated for a *Bacillus subtilis* (*B. subtilis*) strain the inhibitory effect of AAA on MK synthesis. Because MKs, as well as AAA, are synthesised via the shikimate pathway, the supplementation with AAA results in a lower MK content due to feedback inhibition. In accordance with this observation, a previous study also showed for *L. monocytogenes* strains the inhibitory effect of AAA on MK-7 synthesis and moreover an effect on the phase transition performance of the membrane-associated with the lowered MK content (Seel et al. 2018). The present study demonstrated the relationship between MK content and bacterial cold adaptation and revealed the regulation of MK synthesis as additional adaptive response to growth under low temperature conditions.

## Materials and methods

### Materials

All chemical reagents and solvents were purchased from Alfa Aesar, Carl Roth, MilliporeSigma, Sigma-Aldrich, Thermo Fisher Scientific, and VWR. Solvents and water for analytics were of HPLC grade and used as received.

### Strains, culture media, and cultivation

In this research, three different strains of *L*. *monocytogenes* were examined. The strain FFH (= DSM 112142; serovar group 4b, 4d, or 4e) was isolated from minced meat in 2011 and the strain FFL 1 (= DSM 112143; serovar group 1/2a or 3a) from Irish smoked organic salmon in 2012. The type strain DSM 20600^T^ (serovar group 1/2a) was included as the reference strain in this study. The type strain was obtained from the Leibniz Institute DSMZ-German Collection of Microorganisms and Cell Cultures. The two strains FFH and FFL 1 were deposited at the DSMZ open collection. These strains were assigned to serovar groups by multiplex polymerase chain reaction (PCR) according to the method of Doumith et al. (2004). The adaptive response of strain FFL 1 to low temperature is primarily a fatty acid-dependent mechanism while strains DSM 20600^T^ and FFH expressed, in addition, MK-mediated response (Seel et al. 2018).

All strains were aerobically cultured in 100 ml tryptic soy broth-yeast extract (TSB-YE) medium composed of tryptic soy broth containing 17.0 g peptone from casein l^−1^, 3.0 g peptone from soy l^−1^, 2.5 g d-glucose l^−1^, 5.0 g sodium chloride l^−1^, and 2.5 g dipotassium hydrogen phosphate l^−1^ supplemented with 6.0 g yeast extract l^−1^ or in 100 ml ultra-high temperature processed milk (UHT-milk, 3.5% fat) using 300 ml Erlenmeyer flasks. The TSB-YE medium or the UHT-milk, respectively, was supplemented with a mixture of the AAA l-phenylalanine, l-tryptophan and l-tyrosine (each of 200, 400, 600, or 800 mg l^−1^). Non-AAA l-alanine, l-cysteine, and l-serine were used as controls. Water activity (*a*_w_) of the medium was measured with a LabMaster-aw instrument (Novasina, Switzerland). Growth in TSB-YE medium was documented by optical density (OD) at 625 nm with a GENESYS 30 visible spectrophotometer (Thermo Fisher Scientific, USA) and fitted by the Gompertz growth model as described by López et al. (2004). Cultures were prepared in two to six independent replicates, inoculated with 1% (vol/vol) of an overnight culture for growth in TSB-YE medium or 0.02% (vol/vol) for cultivation in milk, and incubated on an orbital shaker at 6 °C and 150 rpm until late exponential phase (OD_625nm_ = 0.8–1.0). Cultures were harvested by centrifugation (10,000×*g* for 10 min) at growth temperature and washed thrice with sterile phosphate-buffered saline (PBS) which was adjusted to growth temperature, pH 7.4. Subsequently, this biomass was used for fatty acid analysis, determination of MK-7 content, and temperature stress test. Colonies were cultivated on tryptic soy agar-yeast extract (TSA-YE) medium at 30 °C.

To determine colony forming units (CFU) for the temperature stress test and the growth inhibition test in milk, 50 µl of serial dilutions were plated on TSA-YE medium (90 mm Petri dish) using the exponential mode (ISO 4833-2, ISO 7218 and AOAC 977.27) of the easySpiral automatic plater (Interscience, France). After a 1-day incubation at 37 °C, the CFU were counted for the corresponding dilution steps and the weighted average of enumerated *L*. *monocytogenes* was given in CFU ml^−1^. The results for the temperature stress test and the growth inhibition test in milk were presented as decadic logarithm (log_10_) decrease or increase relative to the initial CFU ml^−1^, respectively.

### Temperature stress tests

Each strain was subjected to three freeze–thaw cycles. Three aliquots of 2 ml cell suspension for each strain were frozen at − 20 °C. After 24, 48 and 72 h cells were thawed for 20 min at room temperature and the number of CFU was determined. The remaining sample volume was refrozen for subsequent freeze–thaw cycles.

### Fatty acid analysis

About 40 mg cells per sample were used for the fatty acid analysis. Fatty acids were extracted and analysed as methyl-esters as described by Sasser (1990) in biological triplicates from independent cultures. Fatty acid methyl esters were identified by gas chromatography–mass spectrometry (GC–MS) with a 7890A gas chromatograph (Agilent Technologies, USA) equipped with a 5% phenyl methyl silicone capillary column coupled with a 5975C mass spectrometer (Agilent Technologies, USA), as previously described by Lipski and Altendorf (1997). Fatty acids analysis was performed with MSD ChemStation software (version E.02.00.493, Agilent Technologies, USA) and identified by their retention times and mass spectra.

The effect of alterations in fatty acid profiles associated with the amino acid supplementation on membrane fluidity was determined by calculation of the weighted average melting temperature (WAMT) according to Seel et al. (2018). The melting temperatures for fatty acids were taken from Knothe and Dunn (2009).

### Isoprenoid quinone analysis

About 30–50 mg cells were extracted with methanol-chloroform (9:5, v/v) as described before by Hu et al. (1999) and Seel et al. (2018) in biological triplicates from independent cultures. Extracts were analysed using a 1260 Infinity Quaternary LC system (Agilent Technologies, USA) equipped with a quaternary pump, an autosampler, a thermo-controlled column compartment, and a diode array detector. Compounds were separated isocratically at 30 °C on a Hypersil™ ODS C18 column (Thermo Fisher, United States) using methanol/diisopropyl ether (9:2, vol/vol) as eluent (flow rate of 1 ml min^−1^). Isoprenoid quinones were detected at 270 and 275 nm and were identified by their absorption spectrum and retention time. The quinones were quantified as vitamin K_1_ equivalents using an external calibration curve and an internal vitamin K_1_ standard. Data acquisition was performed with OpenLAB CDS ChemStation software (version C.01.07, Agilent Technologies, USA).

### Statistical evaluation

Statistical analysis was performed using Prism (version 8.4.3; GraphPad Software, United States). Mean values and standard deviations of *n* (see legends) biological replicates were calculated for all experiments. All datasets were tested for normal distribution by Shapiro–Wilk normality test using the method of Royston (1995) before two-way ANOVA with Tukey–Kramer post hoc test (*α* = 0.001) was performed. Potential outlier values were determined using Grubbs outlier tests (Grubbs 1950). Data are presented as means ± standard deviation; *P < 0.001, **P < 0.0001, ***P < 0.00001, ****P < 0.000001.

## Results and discussion

### Menaquinone content affects growth rates and resistance to temperature stress

Several strains of *L*. *monocytogenes* have been described previously, which showed a less significant adaptation of the fatty acid profiles at 4 and 6 °C growth temperature than the majority of *L. monocytogenes* strains (Neunlist et al. 2005; Seel et al. 2018). In accordance with previous findings by Seel et al. (2018), *L*. *monocytogenes* strains DSM 20600^T^ and FFH showed less adaptation in their fatty acid profiles to 6 °C growth temperature based on the ΔWAMT values but had significantly higher MK-7 contents than strain FFL 1 (Table 1). This indicated the involvement of this neutral lipid in the adaptation of membrane fluidity. Although an impact of MK-7 content on the membrane transition phase was reported before, a direct influence of MK-7 on the cold resistance of *L*. *monocytogenes* strains was not analysed so far. To determine whether the suppression of the MK-7 content in *L*. *monocytogenes* affects resistance to temperature stress and growth, studies were conducted by supplementation with AAA, which caused product inhibition of the 3-deoxy-d-arabino-heptulosonic acid 7-phosphate synthase in the shikimate pathway. This approach has been used by Tsukamoto et al. (2001) to reduce the MK content in *B. subtilis*. Supplementation with AAA and non-AAA did not affect the *a*_w_ of the medium (data not shown). The non-AAA l-alanine, l-cysteine and l-serine were used as controls. These non-AAA have similar polarities as l-phenylalanine, l-tryptophan, and l-tyrosine, and were used to exclude cryo-protective properties of amino acids.

**Table 1:** Fatty acid (FA) composition, weighted-average melting temperature (WAMT) and menaquinone-7 (MK-7) content of *Listeria monocytogenes* strains DSM 20600^T^, FFH and FFL 1 grown at 6 °C in tryptic soy broth-yeast extract medium supplemented with 800 mg each of l-phenylalanine, l-tryptophan, and l-tyrosine l^−1^ (AAA), and supplemented with 800 mg each of l-alanine, l-cysteine, and l-serine l^−1^ (non-AAA)

Parameter	DSM 20600^T^		FFH		FFL 1	
	AAA	Non-AAA	AAA	Non-AAA	AAA	Non-AAA
FA composition (%)						
*iso-C*_14:0_	2.2 ± 0.6	2.0 ± 0.1	1.3 ± 0.2	1.7 ± 0.8	1.7 ± 0.9	1.5 ± 0.1
C_14:0_	1.0 ± 0.2	0.7 ± 0.2	0.8 ± 0.4	1.1 ± 0.7	1.2 ± 0.1	0.6 ± 0.1
*iso-*C_15:0_	10.7 ± 1.0	11.5 ± 0.3	8.7 ± 1.0	9.7 ± 1.2	8.3 ± 1.7	8.0 ± 0.0
*anteiso-*C_15:0_	74.8 ± 2.5	74.1 ± 2.1	79.8 ± 1.7	79.2 ± 2.2	84.2 ± 3.6	85.7 ± 0.8
*iso-*C_16:0_	1.4 ± 0.1	1.5 ± 0.1	1.8 ± 1.0	1.6 ± 0.4	1.5 ± 0.3	2.0 ± 0.5
C_16:0_	0.6 ± 0.4	1.6 ± 1.2	1.3 ± 0.7	1.3 ± 0.4	0.5 ± 0.2	0.6 ± 0.3
*iso-C*_17:0_	0.5 ± 0.1	0.4 ± 0.1	0.5 ± 0.1	0.5 ± 0.1	0.3 ± 0.1	0.3 ± 0.0
*anteiso-C*_17:0_	8.7 ± 1.2	8.3 ± 0.1	5.9 ± 0.6	5.0 ± 0.2	2.3 ± 0.9	1.3 ± 0.1
WAMT (°C)	30.0 ± 1.6	30.4 ± 1.2	29.1 ± 1.8	29.4 ± 1.8	28.3 ± 2.1	28.1 ± 0.6
MK-7 (nmol g^−1^_cell wet wt_)	125 ± 14****	204 ± 5	94 ± 18****	165 ± 12	77 ± 7	85 ± 15

Values are means ± standard deviation (*n* = 3). Asterisks represent *P* values, (****P < 0.000001) between cultures supplemented with AAA and with non-AAA

**Fig. 1:**
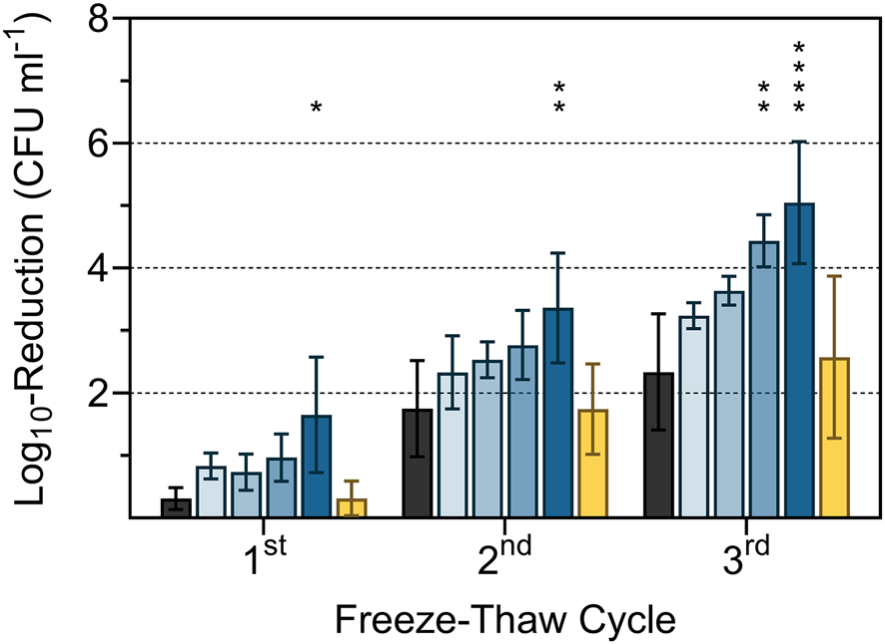
Logarithmic reduction of viable cell counts of *Listeria monocytogenes* strain FFH grown at 6 °C in tryptic soy broth-yeast extract medium without supplementation (black), with 200, 400, 600, and 800 mg each of l-phenylalanine, l-tryptophan, and l-tyrosine l^−1^ (AAA; very light blue, light blue, blue, dark blue), and with 800 mg each of l-alanine, l-cysteine, and l-serine l^−1^ (non-AAA; yellow) after one, two and three freeze–thaw cycles (each 24 h) relative to the initial cell count. Values are means ± standard deviation (very light blue, light blue, and blue *n* = 3; black, dark blue, and yellow *n* = 6). Asterisks represent *P* values (*P < 0.001, **P < 0.0001, ***P < 0.00001, ****P < 0.000001) between cultures supplemented with AAA and with non-AAA as well as without supplementation

**Fig. 2:**
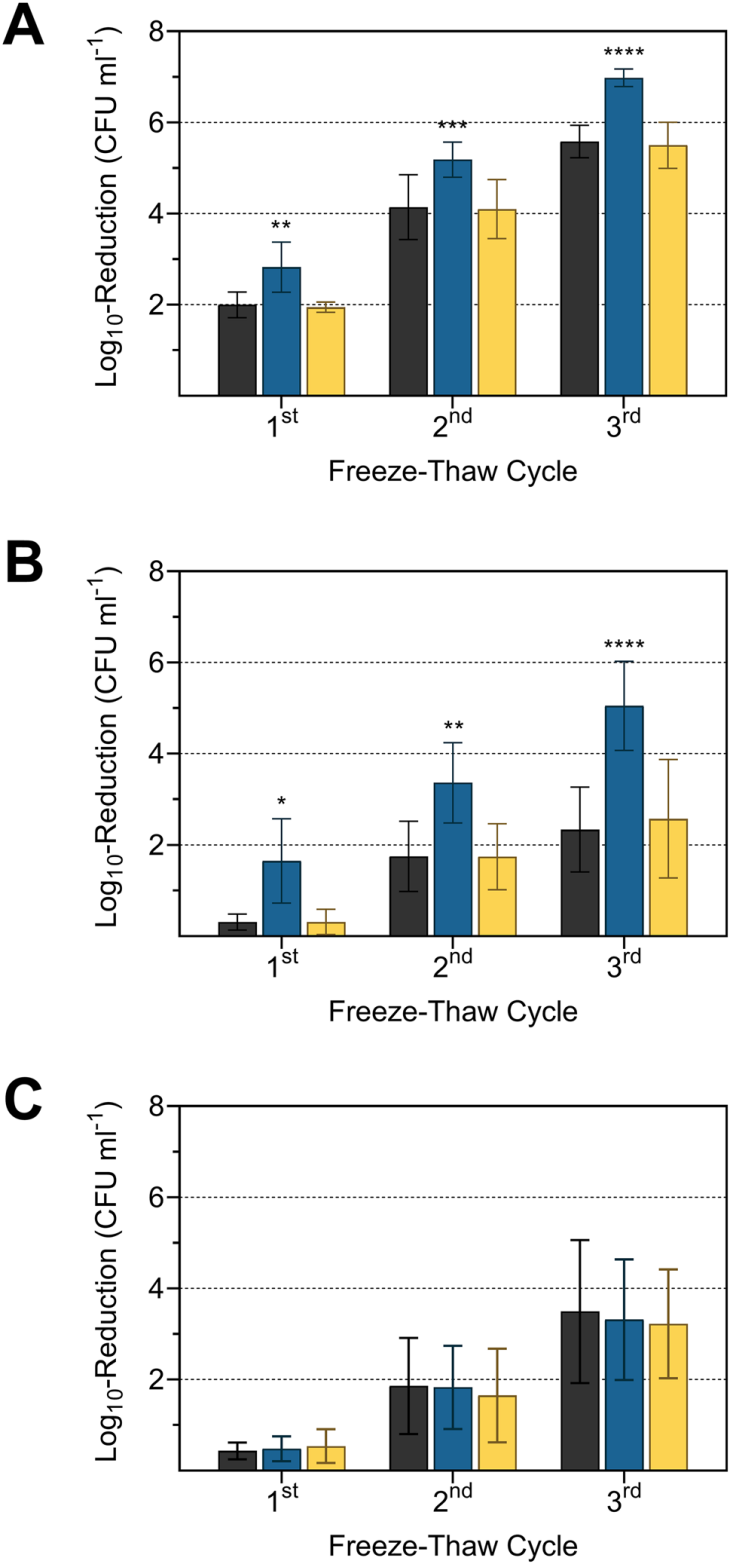
Logarithmic reduction of viable cell counts of *Listeria monocytogenes* strains DSM 20600^T^ (**a**), FFH (**b**) and FFL 1 (**c**) grown at 6 °C in tryptic soy broth-yeast extract medium without supplementation (black), with 800 mg each of l-phenylalanine, l-tryptophan, and l-tyrosine l^−1^ (AAA; dark blue) and with 800 mg each of l-alanine, l-cysteine, and l-serine l^−1^ (non-AAA; yellow) after one, two and three freeze–thaw cycles (each 24 h) relative to the initial cell count. Values are means ± standard deviation (*n* = 6). Asterisks represent *P* values (*P < 0.001, **P < 0.0001, ***P < 0.00001, ****P < 0.000001) between cultures supplemented with AAA and with non-AAA as well as without supplementation

**Fig. 3:**
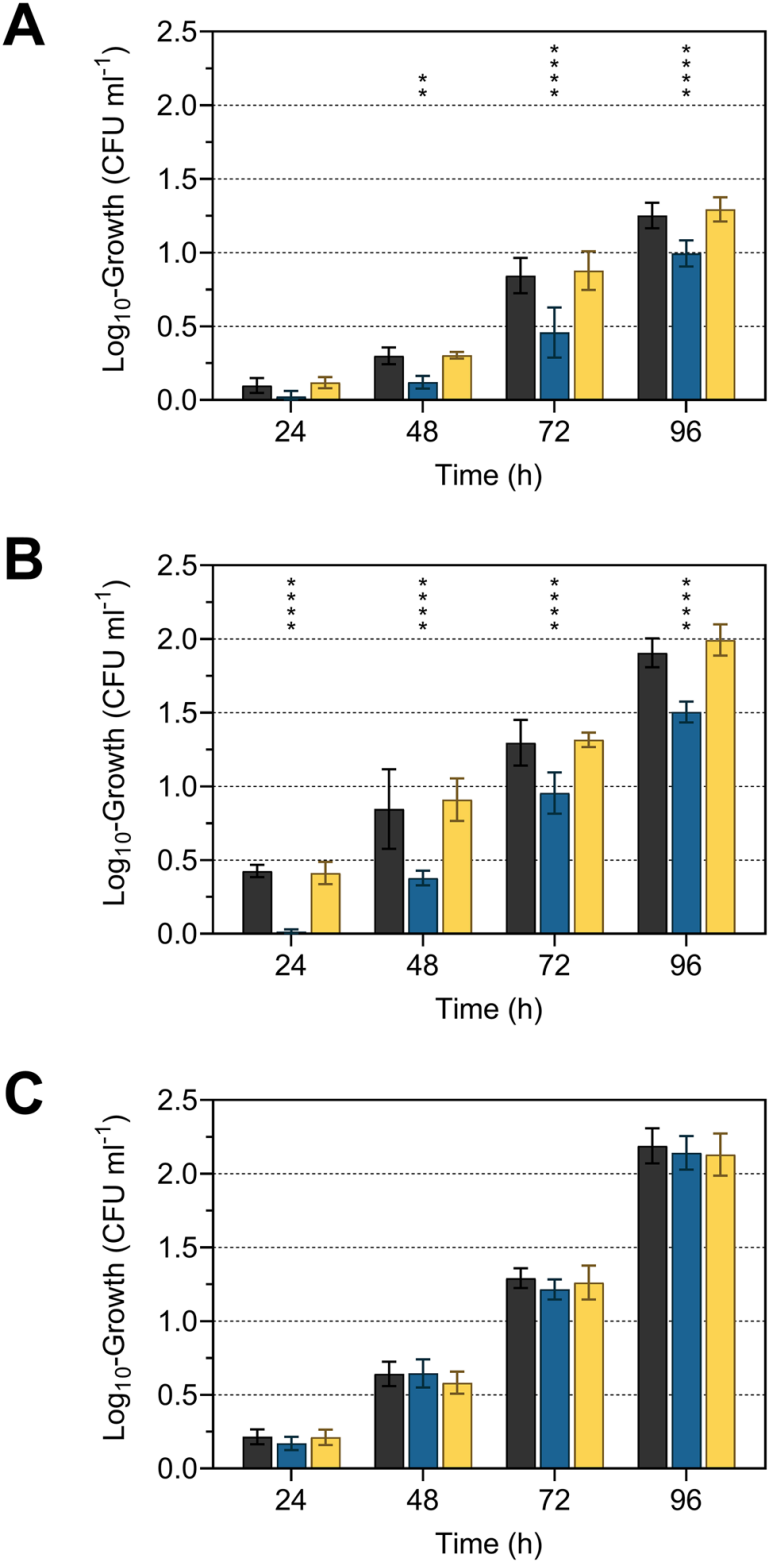
Logarithmic growth of *Listeria monocytogenes* strains DSM 20600^T^ (**a**), FFH (**b**) and FFL 1 (**c**) at 6 °C in ultra-high temperature processed milk without supplementation (black), with 800 mg each of l-phenylalanine, l-tryptophan, and l-tyrosine l^−1^ (AAA; dark blue) and with 800 mg each of l-alanine, l-cysteine, and l-serine l^−1^ (non-AAA; yellow) after 24, 48, 72, and 96 h relative to the initial cell count. Values are means ± standard deviation (*n* = 6). Asterisks represent *P* values (*P < 0.001, **P < 0.0001, ***P < 0.00001, ****P < 0.000001) between cultures supplemented with AAA and with non-AAA as well as without supplementation

**Fig. 4:**
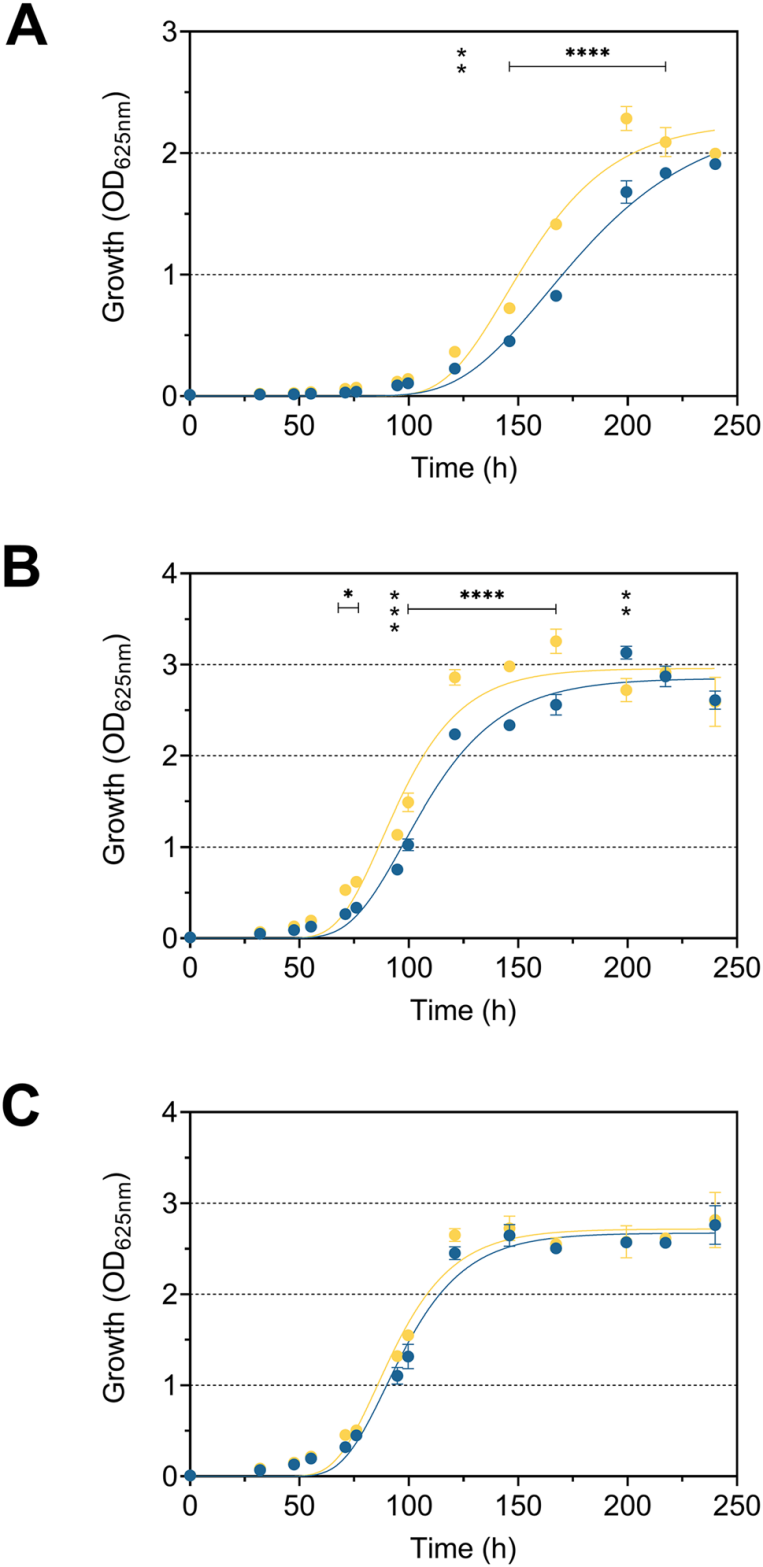
Growth kinetics of *Listeria monocytogenes* strains DSM 20600^T^ (**a**), FFH (**b**) and FFL 1 (**c**) at 6 °C in tryptic soy broth-yeast extract medium supplemented with 800 mg each of l-phenylalanine, l-tryptophan, and l-tyrosine l^−1^ (AAA; dark blue) and with 800 mg each of l-alanine, l-cysteine, and l-serine l^−1^ (non-AAA; yellow). Data are fitted by Gompertz growth model. Values are means ± standard deviation (*n* = 2). Asterisks represent *P* values (*P < 0.001, **P < 0.0001, ***P < 0.00001, ****P < 0.000001) between cultures supplemented with AAA and with non-AAA

To confirm the correlation between MK-7 content and resistance to freeze–thaw stress, MK-7 synthesis in strain FFH was inhibited by adding different concentrations of AAA, ranging from 200 to 800 mg l^−1^ (Fig. 1). After supplementation with AAA, concentration-dependent log_10_-reduction rates were observed, but this was significant only at 800 mg of each AAA l^−1^ after each freeze–thaw cycle. Supplementation with 600 mg of each AAA l^−1^ showed significance only in the third freeze–thaw cycle in strain FFH. This indicates that the feedback inhibition increases with increasing AAA concentrations but is already effective at lower concentrations of AAA. A reduction of the MK-7 content was already achieved with 180 mg of each AAA l^−1^ by Seel et al. (2018). The high concentration with 800 mg of each AAA l^−1^ was used to achieve a more pronounced reduction of the MK-7 content. The freeze–thaw test confirmed the positive influence of MK-7 on bacterial cell resistance to freeze–thaw stress (Fig. 2). After growth at 6 °C and subjected to freeze–thaw stress, *L*. *monocytogenes* strains DSM 20600^T^ and FFH showed a significant reduction of viable cells, quantified as CFU ml^−1^, if supplemented with AAA but were unaffected if supplemented with non-AAA or without amino acid supplementation (Fig. 2). The number of viable cells was significantly reduced after freeze–thaw stress by 2.8 ± 0.6, 5.2 ± 0.4, and 7.0 ± 0.2 log_10_ CFU ml^−1^ for strain DSM 20600^T^ and 1.7 ± 0.9, 3.4 ± 0.9, and 5.1 ± 1.0 log_10_ CFU ml^−1^ for strain FFH after each of the three freeze–thaw steps. This corresponded to a percentage increase in the log_10_-reduction of CFU ml^−1^ from freeze–thaw cycle to cycle compared to the cultures without supplementation of about 41, 25 and 25% and 433, 92 and 116% for strains DSM 20600^T^ and FFH, respectively. No significant increase in the reduction of CFU ml^−1^ could be detected for strain FFL 1 after supplementation with AAA in comparison to non-AAA supplementation and without supplementation, respectively. This is in accord with the absence of any MK accumulation at 6 °C for this strain (Table 1). Moreover, this confirmed the beneficial effect of MK-7 on cell membrane integrity at 6 °C in strains DSM 20600^T^ and FFH. Both strains increase MK-7 content at low growth temperatures. The reduction of MK-7 by supplementation with AAA resulted in a steeper phase transition of the membrane as described earlier by Seel et al. (2018), which resulted in increased susceptibility to freeze–thaw stress as demonstrated in this study. Furthermore, the influence of MK-7 content on the growth characteristics of *L*. *monocytogenes* strains was analysed to detect growth inhibitory effects provoked by AAA supplementation. In addition to TSB-YE medium, milk was used as a growth medium to demonstrate AAA-associated effects also in a food matrix typical for *L. monocytogenes* (Buyser et al. 2001; Fleming et al. 1985). Bacterial cell growth in AAA-supplemented milk was reduced for strains DSM 20600^T^ and FFH after 96 h (Fig. 3). Significant growth inhibition in milk occurred after 24 h in strain DSM 20600^T^ and after 48 h in strain FFH. No growth inhibition was observed for strain FFL 1. Cultures with non-AAA supplementation and without amino acid supplementation showed similar cell growth in milk (Fig. 3). Moreover, growth experiments in TSB-YE medium showed the same significant effects as in milk when AAA were added (Fig. 4). The growth rate was reduced in strains DSM 20600^T^ and FFH after supplementation with AAA compared to the supplementation with non-AAA. The growth rate decreased from 0.036 to 0.026 and 0.050 to 0.041 after supplementation with AAA in strain DSM 20600^T^ and FFH, respectively.

### Membrane adaptation by isoprenoid quinone content substitutes fatty acid profile modification

For all three *L*. *monocytogenes* strains, fatty acid profiles were determined after growth in TSB-YE medium with or without supplementation at 6 °C growth temperature to exclude effects of AAA on them. All three strains showed an *iso*/*anteiso* fatty acid profile with dominating fatty acids *iso*-C_15:0_, *anteiso*-C_15:0_, and *anteiso*-C_17:0_ (Table 1). These three branched-chain fatty acids represented at least 90% of the total fatty acids at 6 °C growth temperature. According to previous findings by Seel et al. (2018), the two strains, DSM 20600^T^ and FFH differed from strain FFL 1 in terms of adaptation of the fatty acid profile to low growth temperatures. Strain FFL 1 showed the highest ratio of *anteiso*-C_15:0_/*anteiso*-C_17:0_, the lowest WAMT value and the lowest MK-7 content at 6 °C growth temperature compared to strains DSM 20600^T^ and FFH. The two strains DSM 20600^T^ and FFH showed two to three times higher content of MK-7 at the growth temperature of 6 °C compared to 37 °C. AAA and non-AAA supplementation did not alter the fatty acid profile in all three strains. Feedback inhibition of MK-7 synthesis was successfully induced by supplementation with AAA in strains DSM 20600^T^ and FFH (Table 1). While both strains showed a significant decrease in MK-7 content after supplementation with AAA at 6 °C growth temperature, strain FFL 1 showed no significant decrease in MK-7 content after AAA supplementation. As expected, no decrease in MK-7 content was detected if cultures were supplemented with non-AAA, which is consistent with the described mechanism for feedback inhibition by Tsukamoto et al. (2001). For all tested *L*. *monocytogenes* strains, only MK-7 was detected. Others, such as MK-5 and MK-6, which had been described for *L*. *monocytogenes* before, were not detected (Collins et al. 1979). The *L*. *monocytogenes* strains DSM 20600^T^ and FFH showed a content of about 204.4 ± 5.4 and 164.7 ± 11.6 nmol MK-7 g^−1^, respectively. Supplementation with AAA reduced MK-7 content by 38.7% in strain DSM 20600^T^ and 42.8% in strain FFH. Strain FFL 1 had a content of about 85.5 ± 14.9 and 76.6 ± 7.3 nmol MK-7 g^−1^ after supplementation with AAA and non-AAA, respectively. The controls with non-AAA of the tested strains are in accord with the MK-7 contents described previously by Seel et al. (2018).

## Conclusion

The disruption of the menaquinone-dependent membrane fluidization under low-temperature conditions resulted in a reduced bacterial cell fitness. This shows that this fatty acid-independent mechanism for regulation of membrane fluidity represents an additional adaptive response to low growth temperatures with a beneficial impact on membrane integrity, growth rate and bacterial cell resistance to temperature stress. The findings suggest that food components such as aromatic amino acids and menaquinone (vitamin K), respectively, may affect growth rates and fitness of certain *Listeria monocytogenes* strains at low temperatures and should be considered for future modelling of food stability against *Listeria monocytogenes* colonization.

## Declarations

### Data availability

The data sets generated and analysed during the current study are available on request from the corresponding author.
